# Prospective multicentre randomised controlled trial to assess the clinical effectiveness of the novel CirrhoCare digital therapeutic management system: a study protocol

**DOI:** 10.1136/bmjopen-2024-098725

**Published:** 2025-07-06

**Authors:** Olivia Greenham, Kohilan Gananandan, Anu Balaji, Konstantin Kazankov, Karen L Thomsen, Jonathan Bamber, Jenny Philip, Anvi Wadke, Zia Sadique, Maruthi Raja, Ravan Boddu, Ravi Kumar, Rajiv Jalan, Rajeshwar P Mookerjee

**Affiliations:** 1 Liver Failure Group, UCL Institute for Liver & Digestive Health, London, UK; 2 CyberLiver Limited, London, UK; 3 Department of Hepatology and Gastroenterology, Aarhus Universitetshospital, Aarhus, Denmark; 4 UCL Institute of Clinical Trials and Methodology, London, UK; 5 Department of Health Economics, London School of Hygiene and Tropical Medicine, London, UK

**Keywords:** Digital Technology, Clinical Trial, Hepatology, Health informatics

## Abstract

**Introduction:**

Liver cirrhosis accounts for over 10 000 deaths in the UK each year with a total loss of 60 000 quality-adjusted life-years. There is a substantial cost to the NHS of £4.5 billion, with new liver-related decompensation events accounting for the majority of this. Following an acute cirrhosis decompensating event, there is a significant risk of hospital readmission with 90-day readmission rates as high as 53%. Current care in the UK is reactive and patients are often only readmitted when they have presented acutely as an emergency with significant decompensation.

**Methods and analysis:**

CirrhoCare is a prospective, multicentre, randomised controlled trial comparing the CirrhoCare management system with standard-of-care for high-risk cirrhosis patients who have been discharged following an admission with acute decompensation. The CirrhoCare management system comprises a novel digital platform for use in a patient’s home, designed to proactively detect the first signs of new decompensation in patients with established cirrhosis, discharged to the community. This enables a clinician to instigate early community-based care or, if needed, to triage the patient for hospital interventions.

214 patients will be recruited to the CirrhoCare trial from at least 12 UK centres. Patients will be randomised on a 1:1 ratio allocation to the CirrhoCare Management System or standard of care. Participants who are randomised to CirrhoCare will receive a CirrhoCare health kit comprising a smart watch, smart phone with enabled SIM (Subscriber Identity Module) network card, blood pressure monitor, weighing scales and thermometer. Participants will take measurements every morning Monday to Friday and will be followed up for 90 days postdischarge.

The primary objective of this study is to assess the clinical effectiveness of the CirrhoCare digital management system. We hypothesise that its early community-based intervention will reduce the number of unplanned hospital interventions and admissions and prevent liver-related complications when compared with standard-of-care management.

**Ethics and dissemination:**

CirrhoCare is a National Institute for Health and Care Research-funded study (NCT06223893). The study has UK Research Ethics Committee and Health Research Authority (HRA) approvals, with approval granted by the HRA and Health and Care Research Wales committee. The results of this study will be published in peer review journals, disseminated at international conferences as well as established Patient and Public Involvement and Engagement networks.

**Trial registration number:**

ISRCTN11380842.

**STRENGTHS AND LIMITATIONS OF THIS STUDY**

- This is a multicentre, prospective, randomised controlled study.
- The study has been powered according to the results of a pilot study, to show clinical effectiveness.
- The inclusion criteria specifically select participants with acute decompensation of cirrhosis, defined by the European-Foundation Consortium Liver Failure Acute Decompensation score, as the population most in need.
- It is not possible for the study to be blinded due to the nature of the intervention and the actionable insights generated by CirrhoCare, to implement management changes.

## Introduction

 Liver cirrhosis significantly increases patients’ morbidity and mortality, accounting for 2.4% of deaths worldwide and over 10 000 deaths in the UK each year.^1^ It is the only major chronic disease where mortality rates are increasing.^2^ Liver disease has a substantial cost not only to the health service but also the wider economy; 62 000 years of working life are lost each year with a cost of £4.5 billion to the NHS (National Helath Service).^3^ Liver-related decompensation events account for the majority of this mortality and cost.^4^

An acute decompensation in a patient with liver cirrhosis is defined as increasing ascites, variceal haemorrhage, overt hepatic encephalopathy, bacterial infection including spontaneous bacterial peritonitis (SBP) or hepatorenal syndrome—acute kidney injury (HRS-AKI)^5^ which often requires a hospital admission. The number of hospital admissions from complications related to liver disease has increased by 50% over the last 10 years with an increment of 22% just in 2023.^6^ Approximately 85% of patients will survive a decompensating event; however, subsequent hospital readmission rates are unacceptably high. 30-day readmission rates following acute decompensation are between 20% and 30%, with 60-day and 90-day readmission rates at 43% and 53%, respectively.^7^,^10^ Bacterial infection, hepatic encephalopathy and ascites have been identified as the main contributing factors for hospital readmission.^11 12^ There is clearly an unmet need to reduce unplanned hospital admissions and create individualised care to initiate early intervention.

Studies have shown that specialist follow-up after discharge is associated with a lower 30-day readmission rate and 12-month mortality.^13^ However, the current standard of care following discharge from hospital for an acute decompensating event is reactive, and significant regional variations exist within our current liver care pathways, leading to regional inequalities, highlighted by the UK Liver Atlas 2017 report.^14^ Patients also often present to hospital late with significant liver-related complications, due to the inadequacies of current care pathways in enabling advanced warning of changes to clinical status in the community. A patient and public involvement (PPI) workshop study preceding our Pilot study highlighted that carers feel poorly supported and are unsure from whom to seek help if a decompensating event occurs. This is in addition to overstretched primary care which struggles to cope with such complex patients. Attempts have been made to identify readmission prediction markers in order to create risk stratification models, but with little success to date.

**Figure 1:**
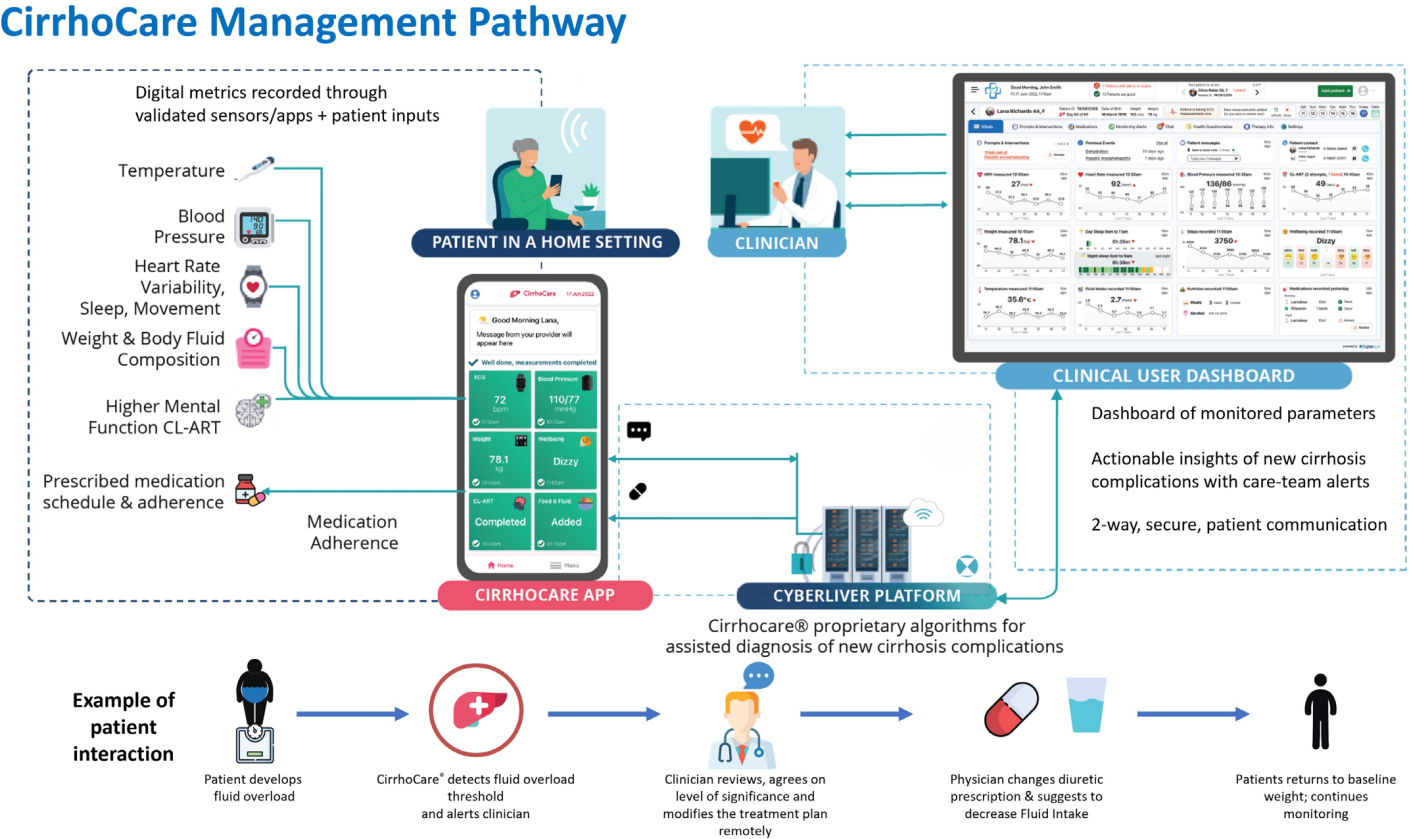
The CirrhoCare management system—a digitally enabled care pathway for patients with decompensated cirrhosis.

The CirrhoCare management system (figure 1) is a novel, patient-centred, home digital therapeutic approach created to address some of these issues. It aims to allow regular patient monitoring via the automatic upload of patients’ physiological measurements, along with daily weights and assessment of hepatic encephalopathy, to a clinician facing digital dashboard with an inbuilt text messaging service. This allows two-way communication between the patient and clinician. The clinician may also choose to speak to the patient via regular telephone calls if required. CirrhoCare aims to identify the early signs of an acute decompensating event, thereby allowing proactive community-based management and prioritisation of early assessment if required, avoiding emergency hospital interventions including use of costly resources.

**Figure 2:**
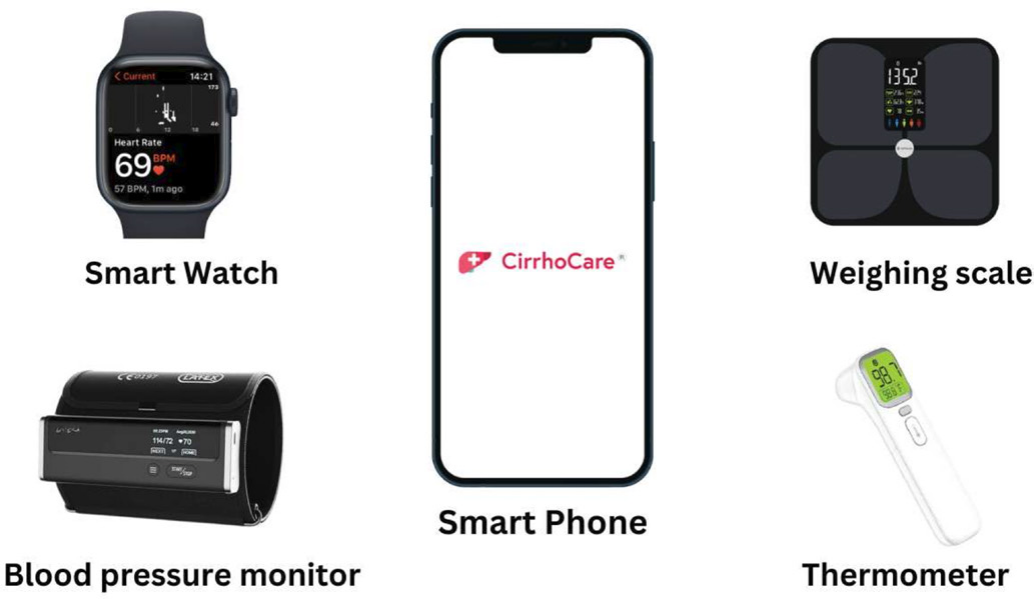
The CirrhoCare healthcare kit.

The CirrhoCare management system works by asking the patient to take a series of measurements using the CirrhoCare healthcare Kit (figure 2), which enables the daily monitoring. The CirrhoCare healthkit comprises a smart watch (heart rate, heart rate variability monitoring, step count and sleep data); smart phone (for engaging with the CirrhoCare App, CL-ART App cognitive testing and patient well-being data collection); a digital Bluetooth-linked blood pressure monitor, bioimpedance scale and thermometer, for physiological measurements. The average time to complete all daily assessments is approximately 12–15 min. These digital sensors are paired by Bluetooth to the CirrhoCare app on the SIM-enabled smartphone, supplied to participants for home monitoring. The digital participant data are transferred to CyberLiver’s platform and secure cloud, analysed by proprietary algorithms and presented as actionable insights on the clinical team’s Dashboard. The two-way participant communication then helps provide proactive community care, by enquiring about signs and symptoms such as changing bowel habit and infective symptoms, which facilitates dose adjustments of standard medications such as diuretics for fluid overload and laxatives for preventing constipation-driven encephalopathy.

A pilot study was conducted in 2020 with 20 patients and 20 controls. The CirrhoCare management system demonstrated clear usability by patients.^15^ This initial study showed a 12-week overall reduction in hospital admissions to 38% in the CirrhoCare treatment arm compared with 60% in the standard-of-care arm. It also showed CirrhoCare led to a reduction in unplanned hospital interventions such as reducing paracentesis by over 80% and also an overall reduction in patient’ cirrhosis severity scores (Model for End-Stage Liver Disease sodium (MELD) and European-Foundation Consortium Liver Failure–Acute Decompensation score (CLIF-C AD)). These promising results have enabled some further artificial intelligence modelling to enhance the diagnostic algorithms that underpin the current version of the CirrhoCare system. This is to be applied in a multicentre randomised controlled trial to assess the clinical and cost-effectiveness of the CirrhoCare digital-therapeutic management system.

We hypothesise that the CirrhoCare management system can reduce liver-related complications requiring unplanned hospital interventions within 90 days following hospital discharge for an acute decompensation of cirrhosis admission, compared with standard-of-care management.

## Methods

### Study design

CirrhoCare is a prospective, multicentre, randomised unblinded controlled trial comparing the CirrhoCare management system with standard of care for high-risk patients with cirrhosis who have been discharged following an admission with acute decompensation. 214 patients with advanced liver cirrhosis will be recruited to the trial from at least 12 sites throughout the UK. The University College London (UCL) Liver Failure Group, UCL Comprehensive Clinical Trials Unit (CCTU) in collaboration with CyberLiver and the London School of Hygiene and Tropical Medicine were involved in the study design. SPIRIT (Guidnace statement for evidence-based recommendations for minimal content of a clinical trial protocol) guidelines were used for the reporting of this protocol.^16^

### Participant selection

The study will include all participants aged 18 years or over with cirrhosis of any aetiology who have been admitted to hospital with an acute decompensating event (increasing ascites, variceal haemorrhage, overt hepatic encephalopathy, SBP or HRS-AKI). Participants must have a CLIF-C AD score^17^ of greater than or equal to 42 and less than or equal to 65 at the time of screening to be eligible. The full inclusion and exclusion criteria are outlined in box 1. Participants will be required to provide written consent prior to recruitment to the trial (online supplemental file 1).

Box 1 **List of inclusion and exclusion criteria**

**Inclusion criteria:**

1. Adults ≥18 years and diagnosed with cirrhosis of any aetiology.
2. Cirrhosis defined by standard clinical criteria, ultrasonographic findings and/or histology. Cirrhosis of any aetiology may be included. However, participants with cirrhosis due to autoimmune hepatitis must be on a stable corticosteroid dose for a ≥3-month period before study inclusion (to be recorded on concomitant log).
3. Cirrhosis severity-risk defined by CLIF-C AD score ≥42 points but ≤65 points at the time of screening.
4. Hospitalisation for acute decompensation (determined as one or more of the following: increasing ascites, variceal haemorrhage, overt hepatic encephalopathy, SBP or HRS-AKI).^5^
5. Participants able to give informed consent.

**Exclusion criteria:**

1. Participants with ACLF (Acute-on-chronic liver failure) grade 2 and above according to the criteria published by Moreau *et al*.^21^
2. Participants with CLIF-C AD score ≥66, who have a high mortality similar to ACLF ≥2 participants.^17^
3. Current overt hepatic encephalopathy, defined as grade II–IV hepatic encephalopathy according to the West-Haven classification,^22^ unable to give consent.
4. Participants with active hepatocellular carcinoma (HCC) or history of HCC that is in remission for less than 6 months for uninodular HCC or for less than 12 months for multinodular HCC within Milan criteria.
5. Participants with a history of significant extrahepatic disease with impaired short-term prognosis, including congestive heart failure New York Heart Association grade III/IV,^23^ COPD (Chronic Obstructive Pulmonary diseas) GOLD >2, chronic kidney disease with serum creatinine >2mg/dL or under renal replacement therapy.
6. Participants with documented refractory ascites on a palliative pathway.
7. Participants who are active on the liver transplant waiting list.
8. Participants with current, active, extrahepatic malignancies including solid tumours and haematologic disorders.
9. Participants with mental incapacity, significant language barrier or any other reason considered by the investigator precluding adequate understanding, cooperation or compliance in the study.
10. Participants with active viral infections or yet to achieve a clear response to antiviral therapy.
11. Any disorders likely to impact on study engagement, including severe frailty, severe addiction history (including opioids) with evidence of multiple recent relapses.
12. Any other reason that the PI (principal investigator) considers would make the participant unsuitable to enter CirrhoCare (eg, participants on an end-of-life palliative care pathway).
13. Participants enrolled in other interventional trials.
  CLIF-C AD, Consortium Liver Failure Acute Decompensation score; HRS-AKI, hepatorenal syndrome–acute kidney injury; SBP, spontaneous bacterial peritonitis.

### Participant recruitment and randomisation

**Figure 3:**
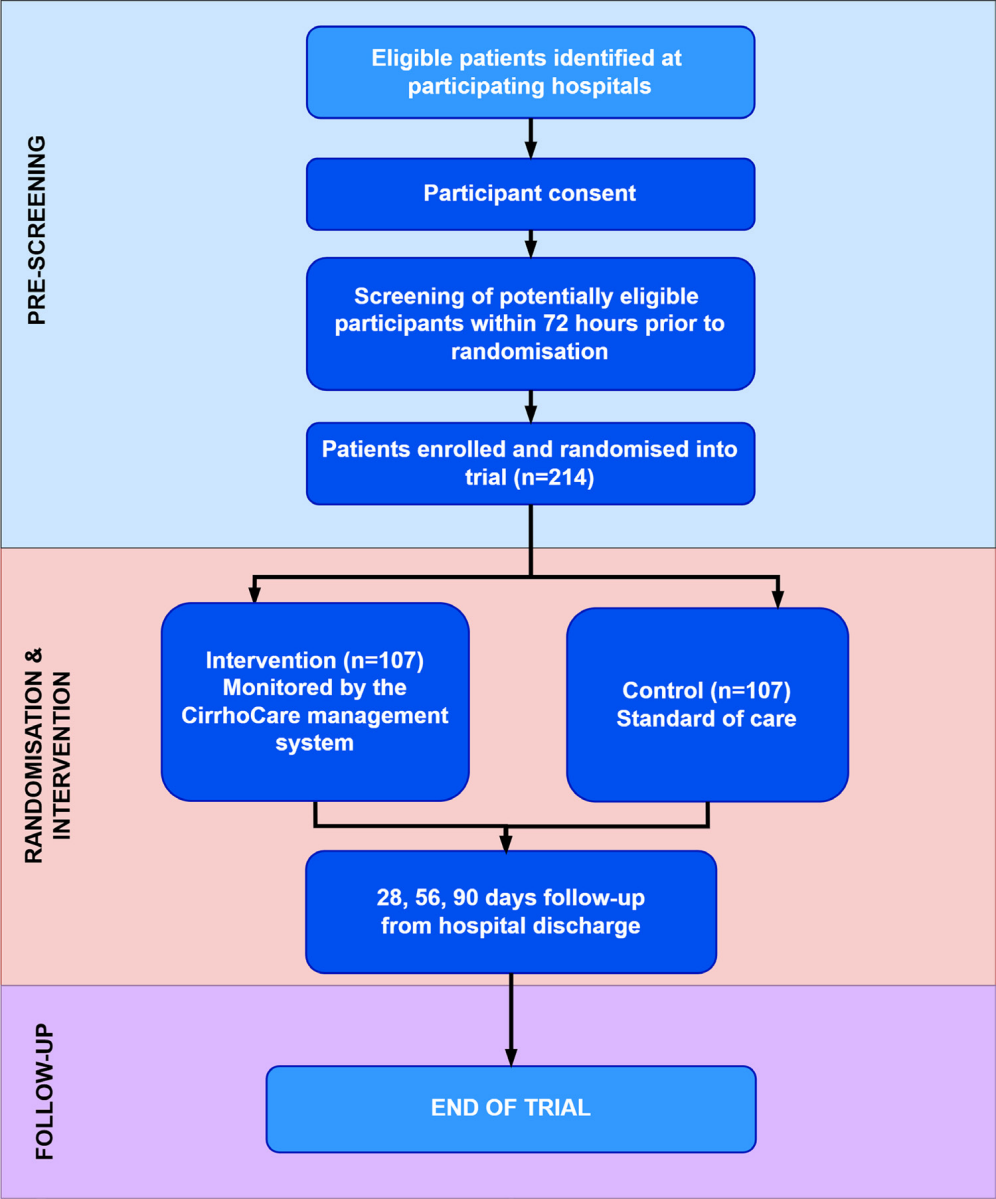
The CirrhoCare study design.

Patients will be recruited ideally prior to, or within a maximum of 14 days postdischarge from hospital. Participants should ideally be randomised within 72 hours of being screened and declared eligible for the trial. Each participant will be randomised on a 1:1 allocation ratio to either the intervention or standard-of-care arm by Sealed Envelope. Each patient will then be followed up for 90 days, including three follow-up time points at 28 days, 56 days and at the end of the trial at 90 days (figure 3). Recruitment has commenced in December 2023; the last participant is now expected to be recruited by June 2026, pending confirmation of an extension of the study timeline from May 2025 as currently stands by the funders, with the final analysis and report submission expected by January 2027.

### Completing measurements

If randomised to the intervention arm, ideally prior to hospital discharge, or within 14 days of discharge, patients will be trained on a CirrhoCare healthcare kit so that they are able to complete their assessments at home. The participants will then be expected to complete their assessments every morning, Monday to Friday. The average time taken to complete a full assessment is 12–15 min. These data will then be analysed by the CirrhoCare clinical research team who will, when required, provide patients with clinical advice including to repeat measurements, to alter medications, to attend an urgent clinical review or the accident and emergency department, either via the text messaging function on the app or by direct clinical contact. In the evening, the patient will complete the medication and dietary/fluid intake log on the CirrhoCare app.

If a participant fails to input their measurements, the research team may send a reminder via the app, or instigate further contact under their discretion, if indicated. If the patient has not completed their measurements for three consecutive days, then the participant will be contacted by a member of the trial team.

### Evaluations during and after treatment

All participants will undergo a baseline visit, usually on the day of discharge, including a full history and examination, physiological parameters, routine blood tests, calculation of MELD, CLIF-C AD scores, liver frailty index (LFI), health-related quality of life (EQ-5D-5L, Euro Qol - quality of life questionnaire used for evaluation of health economics and effectiveness of a therapy) questionnaire, and a record of concomitant medication. These parameters will be assessed at subsequent time points during face-to-face follow-ups at 28 days and 90 days. There will also be a contact point with a telephone follow-up at 56 days.

### Sample size

Data obtained from the CirrhoCare pilot study informed a baseline event rate of 35% compared with 60% for standard of care. We used Fisher-Irwin’s exact conditional test and aimed for 90% power (1−beta) to detect a difference between arms with a 5% two-sided alpha rate. Additionally, we adjusted the sample size to allow for 15% loss to follow-up or methodological challenges. This resulted in the trial requiring 214 participants to be randomised 1:1 to the two allocated arms, in order to observe 182 evaluable participants in total.

### Consent

Participants must be able to provide written informed consent (online supplemental file 1) at the time of recruitment to be eligible for the study. The participant will be provided with a participant information sheet and be given sufficient time to read this and ask questions regarding the trial. If a patient loses capacity during the trial (eg, due to developing overt HE), then a nominated professional consultee will be approached to advise if it is in the patient’s best interest to continue with the trial.

### Study intervention

The CirrhoCare management system is a UK Conformity Assessed-marked, Digital Technology Assessment Criteria compliant, digital-therapeutic-system. It consists of high-fidelity cirrhosis monitoring sensors and a smartphone app; a clinician-facing, decision-facilitating dashboard and CyberLiver’s platform incorporating proprietary algorithms (figure 2).

### Patient and public involvement

The project has benefited from public and patient involvement from the outset, with many design changes and informed learning, implemented after the pilot trial, and with a PPI workshop and feedback questionnaire undertaken. Moreover, the educational tools have benefited from the oversight of our PPI and engagement (PPIE) group associated with the project.

Richard Allen, CirrhoCare PPIE co-lead, has provided insights on the information generated for patients for the CirrhoCare trial, in addition to educational resources for use on the CirrhoCare app.

A summary of trial results will be made available to all participants at the end of the study, and if the participant is in the intervention arm, they will receive real-time feedback on their results throughout the study.

### Monitoring

The Independent Data Monitoring Committee (IDMC) is an independent body and not involved in site recruitment, nor linked with the sponsor or with any competing interests.

No interim analysis is planned, and any decision to terminate the trial will be via the IDMC and discussed with the trial steering committee and sponsor.

All adverse events, whether expected or not, will be recorded in the participant’s medical notes and, if trial related, reported to the sponsor via the relevant case report forms within 10 days. Events that meet the definition of a serious adverse device effect will be required to be reported to the sponsor within 24 hours.

Any trial auditing will be independent of the investigators but overseen by the trial sponsor. There will be an independent statistician and clinical adjudication committee who will evaluate the data outcomes and audit the clinical interactions, respectively.

## Analysis

### Outcomes

#### Primary objectives

The primary objective of this study will be to assess the clinical effectiveness of CirrhoCare’s digital therapeutic management system.

#### Primary endpoints

The primary endpoint of this study will determine whether, following hospital discharge after an acute decompensation admission, the CirrhoCare management system leads to a reduction in unplanned medical interventions for new-liver related complications, within 90 days from hospital discharge. Planned interventions are defined as scheduled appointments as part of the patient’s standard of care, or any intervention initiated by a liver specialist (consultant, specialist trainee or prescribing nurse practitioner). This includes but is not limited to scheduled admissions, clinic appointments, day-case procedures and interventions and actions facilitated by the CirrhoCare system. All other medical interventions should be considered unplanned.

New liver-related complications are defined as:

1. Overt encephalopathy (defined by a grade ≥2 according to the West Haven criteria).^17^
2. Dehydration (defined by either AKI criteria according to the International Club of Ascites)^18^:
  - Creatinine greater than 1.5 times baseline.
  - Creatinine increase >0.3 mg/dL or >26.5 umol/L within 24–48 hours.
3. Increasing ascites (defined as large volume grade 3 ascites).
4. Variceal bleeding.
5. Infection—defined by the following:
  - Presence of positive cultures from urine, ascites, sputum or blood, or radiological chest criteria, or SBP with polymorphonuclear counts >250 cells/mm^3^.
  - High clinical suspicion of infection warranting inpatient, pre-emptive antimicrobial treatment.

#### Secondary objectives

The secondary objectives of the study will be to assess the effect that CirrhoCare has on the individual components of the primary outcome over the 90-day follow-up period, including effect on severity of liver disease (using liver disease severity scores CLIF-C AD or MELD); effect on length of hospital stay (including intensive care admission), hospital readmissions and liver-related and overall mortality. The study will also aim to assess qualitative measures such as user experience, effects on quality of life (EQ-5D-5L) and frailty assessment (LFI).

Crucially, while the main objective is to determine the clinical effectiveness of the CirrhoCare programme, a formal cost-effectiveness analysis will be conducted to determine differences in healthcare costs between CirrhoCare and standard-of-care arms. Furthermore, a full work package of qualitative research will be undertaken, using questionnaires and interviews to identify barriers to implementing CirrhoCare in real-world clinical practice.

#### Data collection and storage

Trial data will be stored in a database created specifically for the CirrhoCare trial hosted by OpenClinica. Data entered into the CirrhoCare’s application will be stored in a secured NHS and General Data Protection Regulation compliant, Amazon Web Services cloud server, based in the UK. The UCL sponsor and CyberLiver will be responsible for data handling, storing, and processing of participant data collected via the app. The UCL sponsor and CyberLiver will follow the principles of the UK DPA.

#### Ethics and dissemination

The study has UK Research Ethics Committee (Health and Care Research Wales Committee) and Health Research Authority approvals. The information pack will be submitted locally to each NHS Trust’s Research and Development office by UCL CCTU. The results of this study will be published in peer-reviewed journals, disseminated at international conferences as well as established PPIE networks. The study has ISRCTN registration (ISRCTN11380842).

## Discussion

CirrhoCare aims to address the care gap that exists through remote digital monitoring and management of cirrhosis, using actionable insights to help diagnose complications early, allowing CirrhoCare to provide timely, cost-effective, community interventions. This also enables specialist liver care in remote geographical locations for integrated care delivery, with additional environmental benefits through fewer hospital journeys. This aligns well with the NHS long-term vision to improve outcomes in chronic conditions, like cirrhosis, through digitally enabled, community-based care.^19^ Furthermore, it enables the delivery of sustainable hepatology care in what is already an overstretched and overburdened healthcare system.

This study also fits within the remit of the National Institute for Health and Care Research Invention for Innovation i4i Challenge programme,^20^ seeking to provide clinical effectiveness data for a new, cost-effective care pathway for cirrhosis, which can be rapidly implemented, given the strides already made in CirrhoCare’s product development and regulatory approvals.

This trial will assess the impact of CirrhoCare, a novel digital therapeutic management system, on the prevention of hospital intervention and readmissions, and the development of new liver-related complications in cirrhosis patients. Furthermore, the study extends to ancillary studies including a nutritional impact analysis and a planned assessment of all the digital biomarkers, to determine if there are correlations with pathophysiology and outcomes, which enable derivation of a new cirrhosis complications prediction tool.

We believe the robust, multifaceted study design, with clinical effectiveness, cost-effectiveness and qualitative arms, will provide valuable insights into the role of digital healthcare in advanced liver disease.

## Supplementary material

10.1136/bmjopen-2024-098725 online supplemental file 1
