# Supplementary material for: Prospective multicentre randomised controlled trial to assess the clinical effectiveness of the novel CirrhoCare digital therapeutic management system: a study protocol
Source: BMJ Open. 2025 Jul 6;15(7):e098725. doi: 10.1136/bmjopen-2024-098725 (PMC12230953; doi:10.1136/bmjopen-2024-098725)
Supplement: online supplemental file 1 [file bmjopen-15-7-s001.pdf]

(To be presented on local headed paper)

CirrhoCare®

UCL COMPREHENSIVE CLINICAL TRIALS UNIT

CirrhoCare Trial: A real-world, randomised controlled study, to determine the clinical and cost-effectiveness of CirrhoCare digital home monitoring and management in patients with decompensated cirrhosis

INFORMED CONSENT FORM

Site Name: **Site Number:**
Name of PI: **Patient ID Number:**

	**Initial boxes to agree**
1. I confirm that I have read and understood the Patient Information Sheet for the CirrhoCare study, version....... dated .... and have been given a copy to keep.	☐
2. I have had the opportunity to ask questions about the study and discuss it with my doctor, and I have received satisfactory answers to all of my questions.	☐
3. I understand that my participation is voluntary and that I can withdraw at any time without giving any reason, without my medical care or legal rights being affected.	☐
4. In the event I withdraw from the study, I am willing for the researchers to continue to use any of my data already acquired.	☐
5. In the event I withdraw from the study, I am willing for the trial to collect data from my medical records	☐
6. I understand that relevant sections of my medical notes and data collected during the study may be looked at by authorised personnel from the Sponsor of the study (University College London), the Comprehensive Clinical Trials Unit (CCTU from UCL), and CirrhoCare Management System providing company (CyberLiver Ltd) as detailed in the patient information sheet, from the Ethics Committee, or from the NHS Trust where it is relevant to my taking part in this research.<br>I agree that data collected about my health care use and medical treatment will be transferred to a collaborating team at London School of Hygiene and Tropical Medical. Your data will be pseudo-anonymised prior to the transfer.<br>I give permission for these individuals to have access to my data/records, but understand that my confidentiality will be maintained.	☐
7. I understand that the results from blood tests done as part of my usual medical care will be checked to confirm eligibility	☐

8. I agree to attend the study appointments at day 28, day 56 and day 90 from discharge.	☐
9. I understand my personally identifiable information will be collected by the CirrhoCare devices (which forms part of the Management System and provided by the company CyberLiver Ltd) in accordance with the Data Protection Act 2018 and confidentiality will be maintained at all times.<br>In addition, if there is a technical issue with the CirrhoCare Management system and devices that I may be contacted directly by the CyberLiver Ltd Information Techenology (IT) team to resolve.	☐
10. I understand that personally identifiable information collected about me may be used by the research team (UCL CCTU) and CyberLiver Ltd for identification and data analysis purposes.	☐
11. I give permission for my personal details to be used to obtain information on my health status during my time in the study. This includes the use of NHS information on subsequent hospital admissions that is held by the relevant NHS body in my country (NHS England (previously NHS Digital)). If I am admitted to hospital while taking part in the study, I consent to the research team, including members of the central study site, to contacting my care providers at that hospital to obtain details relevant to the study, whether that hospital is taking part in the research study or not. I consent to the disclosure of relevant information and medical records by staff at that hospital.	☐
12. I give permission for the research team to consult a nominated professional consultee who is independent from the research team to advise on my behalf if it is in my best interest to continue to participate on the trial in the event I lose my mental capacity during the trial.	☐
13. I give permission for my General Practitioner (GP) to be informed of my participation in the study and to be contacted if necessary to obtain information needed for follow-up.	☐
14. I agree that the research team may contact my next-of-kin or carer to obtain information about my health prior to and during my time in the study.	☐
15. I give consent for the excess blood samples taken as part of routine care, to be transferred and stored at the Liver Failure Group freezers at the Royal Free Hospital headed by Professor Rajeshwar Mookerjee, for ethically approved ancillary study associated with this trial.<br>For sites who are not participating in this aspect of the trial, please write N/A	☐
16. I give consent for the research team to use the clinical information from microbiological rectal swabs performed routinely when an inpatient, and repeated at 90 days, to assess for antimicrobial resistance patterns.<br>For sites who are not participating in this aspect of the trial, please write N/A	

17. I understand that I may not benefit directly by participating in this study but that the research may help people who have decompensated cirrhosis in the future.	☐
18. I agree to share my study data (but not my name) with other researchers, including in the UK and internationally.	☐
19. **(optional)** I would like to be informed of the results of the this study. *Please provide the research team your email address:* ________________________________	☐
20. I agree to take part in the CirrhoCare study.	☐

**Name of Patient (BLOCK CAPITALS)**	**Date (dd/mmm/yyyy)**	**Signature**
**Name of witness (if required) (BLOCK CAPITALS)**	**Date (dd/mmm/yyyy)**	**Signature**
**Name of Researcher taking consent (BLOCK CAPITALS)**	**Date (dd/mmm/yyyy)**	**Signature**

When completed: Original for the local investigator site file, 1 copy for the hospital notes, and 1 copy for the patient.
