# Supplementary material for: Prospective multicentre randomised controlled trial to assess the clinical effectiveness of the novel CirrhoCare digital therapeutic management system: a study protocol
Source: BMJ Open. 2025 Jul 6;15(7):e098725. doi: 10.1136/bmjopen-2024-098725 (PMC12230953; doi:10.1136/bmjopen-2024-098725)
Supplement: Supplementary file 2 [file bmjopen-2024-098725.reviewer_comments.pdf]

PEER REVIEW HISTORY

BMJ Open publishes all reviews undertaken for accepted manuscripts. Reviewers are asked to complete a checklist review form and are provided with free text boxes to elaborate on their assessment. These free text comments are reproduced below.

ARTICLE DETAILS

Title (Provisional)

A prospective multicentre randomised controlled trial to assess the clinical effectiveness of the novel CirrhoCare digital therapeutic management system: Study Protocol

Authors

Greenham, Olivia; Gananandan, Kohilan; Balaji, Anu; Kazankov, Konstantin; Thomsen, Karen L; Bamber, Jonathan; Philip, Jenny; Wadke, Anvi; Sadique, Zia; Raja, Maruthi; Boddu, Ravan; Kumar, Ravi; Jalan, Rajiv; Mookerjee, Raj P.

This article was not externally reviewed at BMJ Open. Protocols that have been independently assessed prior to submission to BMJ Open are usually fast-tracked to publication on the grounds that further substantial changes will not be possible. This independent assessment will usually be external, independent review for both a substantial grant award from a non-commercial or government funder and ethics approval.
